# Phenols Extraction from Sorghum Byproducts: Upcycling Strategies and Food Applications

**DOI:** 10.3390/antiox14060668

**Published:** 2025-05-30

**Authors:** Tiziana Amoriello, Francesco Mellara, Roberto Ciorba, Danilo Ceccarelli, Monica Amoriello, Federica Taddei, Roberto Ciccoritti

**Affiliations:** 1 Consiglio per la Ricerca in Agricoltura e l’Analisi dell’Economia Agraria, Research Centre for Food and Nutrition, Via Ardeatina 546, 00178 Rome, Italy; francesco.mellara@crea.gov.it; 2 Consiglio per la Ricerca in Agricoltura e l’Analisi dell’Economia Agraria, Research Centre for Olive, Citrus and Tree Fruit, Via di Fioranello 52, 00134 Rome, Italy; roberto.ciorba@crea.gov.it (R.C.); danilo.ceccarelli@crea.gov.it (D.C.); 3 Consiglio per la Ricerca in Agricoltura e l’Analisi dell’Economia Agraria, Central Administration, Via Archimede 59, 00197 Rome, Italy; monica.amoriello@crea.gov.it; 4 Consiglio per la Ricerca in Agricoltura e l’Analisi dell’Economia Agrarian, Research Centre for Engineering and Agro-Food Processing, Via Manziana 30, 00189 Rome, Italy; federica.taddei@crea.gov.it

**Keywords:** ultrasound-assisted extraction, response surface methodology, phenolic profile, antioxidant activity, bread, shelf life, hyperspectral imaging, sensorial analysis, sorghum, solvents tested

## Abstract

In this research, ultrasound-assisted extraction (UAE) coupled with response surface methodology was used to identify the optimal process parameters (temperature, time, and solvent composition (ethanol–water ratio)) for bioactive compounds extraction from stalks and debranning products. Extraction efficiency was assessed in terms of total phenolic compounds (TPCs) and antioxidant activity (AA), and phenolic profiles were identified using HPLC-DAD for the four samples of byproducts (two samples from stalks, C1 and C2, and two samples for debranning products, C3 and C4, from two different farms). The optimized extract containing the highest amount of TPC at different levels was used to enrich bread to evaluate the ability of extending their shelf life using a hyperspectral imaging device (935–1720 nm). Under the optimal conditions, the amounts of phenolics on average in stalk increased by about 79.5% and 47.0% in debranning products, whereas the relative AA increased by about 28.5% (C1 and C2 samples) and 63.0% (C3 and C4 samples) when UAE was applied with respect to the traditional technique. The characterization of stalk phenolic acids profile revealed that gallic, ferulic, and 4-coumaric acids were the prevalent ones. Otherwise, caffeic, syringic, and 3-coumaric acids were the most abundant in debranning products. As expected, enriched bread showed a significant increase in TPC and AA values without influence on organoleptic characteristics. Finally, enriched bread showed a shelf-life increase of about five days.

## 1. Introduction

Sorghum, (*Sorghum bicolor* L.), is a cereal crop belonging to the Poaceae (Gramineae) family. It originated in Africa, and later spread widely through Asia, Australia, and America, due to its high adaptability to various climate and soil conditions, its high resistance to drought and salinity, low fertilizing rate, and high resistance to biotic and abiotic stress [1,2,3].

It is the fifth most important cereal crop worldwide, with a wide range of applications in the food, feed, fuel, and industrial sectors. Its adaptability and nutritional profile make it valuable for both human consumption and agricultural systems worldwide [2]. Correspondingly, sorghum can be diversified into four major categories, encompassing grain sorghum, sweet sorghum, forage sorghum, and biomass sorghum [2]. From a human food point of view, sorghum can be used for whole grain consumption, flatbreads and porridges, gluten-free products, malting and brewing, as well as syrup preparation [4]. Sorghum is a major component of livestock feed, especially in semi-arid regions where maize is less productive. The grain can be used in feed rations for poultry, swine, and cattle. Forage sorghums are cultivated for grazing, hay, or silage. Moreover, stover can be used as roughage for ruminants [5]. Sorghum has gained importance as a bioenergy crop, especially in the context of renewable energy and climate resilience [6]. Ethanol can be produced from high biomass and sugar-rich varieties (e.g., sweet sorghum) [7]. Sorghum is used in biogas plants and for combustion in biomass energy systems. Finally, sorghum can be used for various non-food industrial applications, such as for the development of bioplastics and packaging, adhesives and paper, and building materials [8]. The adaptability to dietary and industrial applications is due to its biochemical composition, which can vary a lot, and it can depend on genotype, environmental conditions (growth location, soil type, and climate), agronomic management, and processing [9]. It is a high-energy cereal primarily composed of complex carbohydrates, mainly starch, which accounts for most of its dry matter. Although starch in grain sorghum varies from 32.0 to 72.5 g 100 g^−1^, it generally contains 20–30% amylose and 70–80% amylopectin [10]. Sorghum is also rich in protein (8−14 g 100 g^−1^), fat (2−5 g 100 g^−1^), insoluble fiber (6−15 g 100 g^−1^), beneficial for digestive health and glycemic control, and bioactive components like vitamin B and fat-soluble vitamins (D, E, and K), minerals (iron, zinc, phosphorus, magnesium, and potassium), and phytochemicals and antioxidants, such as anthocyanins and polyphenols [2]. Therefore, sorghum can contribute to human health, due to its antioxidative, anti-inflammatory, and anticancer effects, as well as its free radical scavenging and high antioxidant activity [11].

The cultivation and the processing of sorghum generate several byproducts, which are not part of the primary harvested grain but have economic, agricultural, or industrial value when properly managed or processed. In particular, stalks are the residues of the aboveground biomass left after grain harvest, including stems and leaves. They are employed for livestock fodder (grazing, hay, or silage), for mulching and soil cover to reduce erosion, or as raw material for bioenergy (e.g., biogas, bioethanol, etc.) [12]. With regard to the processed waste, the outer layers of the sorghum grain that are removed during debranning are defined as debranning products. They can be used as animal feed, being rich in fiber and residual nutrients, as dietary fiber supplement in human nutrition, or as a source of antioxidants (especially phenolic compounds) [13]. Other types of waste can be the germ of grain, used as protein-rich feed ingredient or for oil extraction; the bagasse, i.e., the fibrous residue left after crushing the stalks of sweet sorghum to extract juice for syrup or ethanol production, used as biofuel, animal bedding or compost, or substrate for mushroom cultivation [14]. Finally, sorghum residues can also be used as soil amendments through composting or incorporation into the soil; biodegradable packaging using fiber composites; substrate for microbial fermentation or enzyme production; or as growth medium in biotech processes [15]. In the last few years, great attention has been paid to the byproducts phenolic compounds’ recovery using green chemistry techniques and chemometric tools such as ultrasound-assisted extraction, coupled with response surface methodology (RSM). Indeed, UAE is considered a green, efficient, and non-thermal extraction technique that uses ultrasonic waves to enhance the recovery of bioactive compounds from plant materials, including agricultural byproducts like sorghum residues [16]. RSM is a statistical and mathematical tool useful to optimize complex processes, such as ultrasound-assisted extraction, by evaluating the relationships between multiple independent variables (process parameters) and one or more response variables (e.g., TPC, AA, etc.) [16].

In this study, stalks and debranning products from sorghum white hybrid were considered to extract polyphenols and to evaluate the antioxidant activity of the extracts. The extraction was carried out using green solvents, ethanol, and water at different ratios, by applying UAE, while RSM was used to optimize the process parameters (solvent composition, extraction time, and extraction temperature). Then, on the optimized extracts, phenolic acids compositions of two types of residues (stalks and debranning products) were analyzed using HPLC-DAD to evaluate the most abundant phenolic acids. The extract with the highest TPC was used to enrich bread in order to assess its effect on the shelf life of bread, considering the antibacterial properties of phenolic compounds (especially due to gallic and ferulic acids). The potential of the extract as a natural preservative and to extend bread shelf life by inhibiting bacterial growth was evaluated using a short-wave infrared (SWIR) hyperspectral imaging (HSI) device (935–1720 nm). Finally, consumers’ sensory perception and acceptability of enriched bread were investigated.

## 2. Materials and Methods

### 2.1. Raw Materials

Sorghum byproducts were obtained by cultivation (stalks) and processing (debranning products) of white hybrid, named Artista, from an organic farm and an integrated farm in Latium, Italy (Table 1). All byproducts were collected at kernel maturation. Entire stalks (length about 1.60 m) were reduced in size, frozen with liquid nitrogen, and then ground with a laboratory mill (Retsch ZM 200, Retsch GmbH 42781 Haan, Retsch-Allee 1-5, Germany) to obtain fine flour with particle size of ≤500 μm. Sorghum grain was oven-dried at 35–36 °C until the moisture content was less than 12 g 100 g^−1^, below which microbial growth is strongly inhibited [16]. Then, grain was debranned, and the resulting debranning products were ground with a laboratory mill (Retsch ZM 200, Retsch GmbH 42781 Haan, Retsch-Allee 1-5, Germany) to obtain fine flour with particle size of ≤500 μm. All samples were divided into two aliquots, the first for traditional analyses and the second one to optimize the ultrasound-assisted extraction.

Commercial soft wheat flour (Petra Srl, Vighizzolo d’Este, PD, Italy), fresh compressed yeast, and salt for bread were purchased from a local market.

### 2.2. Proximate Composition

Proximate composition of raw materials was performed in triplicate and the data were expressed as g 100 g^−1^ on a dry weight basis (DW). Moisture, ashes, and proteins were determined by the ICC standard methods 110/1, 104/1, and 105/2, respectively [17]. Protein content was estimated using the nitrogen-to protein conversion factors of 6.25 for sorghum byproducts and 5.7 for wheat flour. Total dietary fiber (TDF) content was obtained using a reagent kit (supelco TDF100A, Merck, Darmstadt, Germany), according to the AOAC method (1990) [18].

### 2.3. Determination of Total Phenolic Content, Flavans, Pigment, and Antioxidant Activity

TPC of raw materials and extracts were measured using the Folin–Ciocalteu method, as reported by Ciccoritti et al. [19]. Results are expressed as mg of gallic acid equivalents (GAE) 100 g^−1^ of fresh weight (FW). The vanillin assay method was applied to total flavans content (TTFC) determination according to Sun et al. [20]. TFC was calculated from a calibration curve, using catechin as a standard and the results were expressed as milligram of catechin equivalents per 100 g of fresh weight (mg CAE 100 g^−1^ FW). Pigments, i.e., chlorophyll a (ChA), chlorophyll b (ChB), and yellow pigment (YPG) were determined according to Dere et al. [21]. ChA and ChB were expressed as micrograms per 100 g of fresh weight (μg 100 g^−1^ FW), whereas YPG as grams per 100 g of fresh weight (g 100 g^−1^ FW).

The antioxidant activity of the extracts was evaluated with 2,2-diphenyl-1-picrylhydrazyl (DPPH) and the data were expressed as milligram of Trolox equivalent per 100 g of fresh weight (mg TE 100 g^−1^ FW) [22]. All determinations were performed in triplicate.

### 2.4. Extraction of Soluble Phenolic Compounds by Sorghum Byproducts Using Ultrasound-Assisted Extraction and Optimization Procedure

Soluble phenolic compounds (hereinafter referred to as phenolic compounds) from the four sorghum byproducts samples were extracted using ultrasound-assisted extraction (UAE) by an ultrasonic bath (ElmasonicS30H, Elma Ultrasonic Technology, Singen, Germany). For each sample, 2.0 g of dried and fine-milled material was diluted with 40 mL of different solvents in a tube, mixed, and sonicated at 37 kHz. A mixture of ethanol and water (with different ratio) was used as green solvent for UAE, whereas acidified methanol: water (80:20, 0.1% HCl) was used for the traditional extraction technique, as previously reported by Iadecola et al. [16]. The heating power was set at 200 W.

A Box–Behnken design (BBD) with three process parameters as independent factors (green solvent composition, extraction time, and extraction temperature) was considered for UAE, with these being the factors that most influence extraction efficiency and allow high yield and phytochemical contents [23]. Each factor was coded at three levels, −1, 0, and +1 (Table 2).

The green solvent composition (X_1_) is an ethanol–water ratio (50–65–80%). Extraction time (X_2_) varied between 40 min and 60 min. Extraction temperature (X_3_) ranged from 70 to 80 °C. The values of factor levels were chosen by previous studies on cereals [14]. The maximum settable temperature of the ultrasound equipment was 80 °C; therefore, it was the maximum value for extraction temperature. Times up to 60 min were set because the pilot study demonstrated that longer extraction times did not increase yields and were not economically sustainable. The design included 15 runs (three at the central point); each run was replicated three times.

At the end of sonication, the tube was cooled in a box with water and ice for 10 min, then centrifuged at 1500× *g* for 20 min at 4 °C. Finally, each sample was filtered using a nylon syringe filter (0.45 μm) and the supernatants were immediately analyzed.

RSM was used to optimize the process parameters to carry out the maximum yield in phenols. The predicted TPC content was carried out using a second-order polynomial equation, as follows:

(1) $$Y_{i}=\beta_{0}+\sum_{i=1}^{3}\beta_{i}X_{i}+\sum_{i=1}^{3}\beta_{ii}X_{i}^{2}+\sum_{ij,i<j}\beta_{ij}X_{i}X_{j}+e_{i}$$

where Y = TPC; X_1_ = green solvent composition (%); X_2_ = extraction time (min); X_3_ = extraction temperature (°C); β_0_ = intercept; β_i_, β_ii_, β_ij_ = linear, quadratic, and interactive coefficients, respectively; e_i_ = error term. Goodness of fit of the model was evaluated by the coefficient of determination R^2^, the adjusted coefficient of determination R_adj_^2^, and the lack of fit value [16]. The optimization procedure was carried out using 3D plots and desirability functions, as described by Iadecola et al. [16].

### 2.5. Optimization of Antioxidant Activity by UAE Extracts

The extracts obtained with BBD (Table 2) were also used to identify the optimal extraction conditions to gain the highest antioxidant activity. For this purpose, the RSM and the optimization procedure were used as described in Section 2.4, considering the antioxidant activity as the response variable.

### 2.6. Profiling of Phenolic Acids by HPLC-DAD

Once extracted, phenolic acids (PAs) were analyzed by RP-HPLC, using a Kinetex C18 (150 × 2.1 mm i.d., 1.7 μm) from Phenomenex (Torrance, CA, USA), placed in a column oven set at 30 °C, and Agilent 1290 Infinity Binary LC with UV–Vis photodiode array detector (DAD G4212A) (Agilent, Milan, Italy), as reported by Amoriello et al. [24]. The detected phenolic acids included gallic, vanillic, 4-coumaric, ferulic, caffeic, syringic, 3-coumaric, and sinapic acids. All standards and sample solutions were analyzed in triplicate, and the content of PAs was expressed as micrograms per milliliter of extracted (μg mL^−1^).

### 2.7. Bread-Making Process

The dough of bread used as control (P1) was made with 1000 g wheat flour (14% moisture basis), 750 g of water, 20 g of compressed baker’s yeast, and 20 g of salt. Bread loaves were produced by replacing part of the water content with extract C1, as C1 had the highest phenols content. Hence, bread samples were produced by replacing part of the water content with 10% (P2) and 20% (P3) of extract. We chose to use the extract from the sample with the highest content of phenols, namely, C1. Therefore, an extract from C1 was produced under optimal extraction conditions. Then, the ethanol was evaporated under nitrogen gas flow, and the obtained dry sample was reconstituted with the same water quantity to recover all bioactive compounds extracted.

The ingredients were mixed for 10 min in a planetary bread mixer (Quick 20 by Sottoriva, Marano, Italy). Once the dough was formed, they were placed in closed plastic containers and left to rise in the fridge at 7 °C for 12 h. Then, the dough was scaled into four equal pieces, inserted into rectangular aluminum bread molds, and placed for 2 h in a fermentation cabinet at 30 °C with 85% relative humidity. At the end, they were baked for 10 min at 220 °C and for 30 min at 200 °C in a convection/steam oven. Baking tests were performed on each bread type by using four replicates.

### 2.8. Color Measurements

Color was measured on bread crumb and crust using a Chroma Meter CR-400 (Konica Minolta, Tokyo, Japan) and (CIELab) L*a*b* scale. The CIELab color space coordinates are the lightness L*, from black 0 to white 100; the redness a* from green (−100) to red (+100); and the yellowness b* from blue (−100) to yellow (+100). The measurements took place on day 1, and the results are the average of measurements of four different points per sample.

### 2.9. Consumer Acceptance

The organoleptic perception was assessed by forty consumer volunteers aged between 20 and 62 years using a consumer test. Approximately 1 cm thick slices of bread for each type of bread were served on white plastic plates. After each tasting, a glass of water was provided for rinsing. Questions about taste, appearance, aroma, color, and overall acceptability of the breads were asked to the consumers. The intensity of each attribute was rated on a 7-point Hedonic scale (1 = dislike very much, 2 = dislike moderately, 3 = dislike slightly, 4 = neither like nor dislike, 5 = like slightly, 6 = like moderately, 7 = like very much), according to Moskowitz [25]. Mean values of all tests for each attribute gave the relative scores.

### 2.10. Evaluation of Raw Materials and Bread Shelf Life by Hyperspectral Images Analysis

The chemical profile of raw materials was analyzed using two different HIS devices: a portable Specim IQ hyperspectral camera (Specim, Spectral Imaging Ltd., Oulu, Finland), operating in the visible and near-infrared (VisNIR) regions between 400 nm and 1000 nm [26], and a SisuCHEMA Hyperspectral Chemical Imaging Analyser (SPECIM, Spectral Imaging Ltd., Oulu, Finland), ranging in the short-wave infrared regions (SWIR: 935–1720 nm) [27]. The shelf life of breads was evaluated using only the SWIR system, monitoring two slices of the three types of breads every day. The bread images acquisition was carried out on the second day (T1), third day (T2), fourth day (T3), and seventh day (T4) after cooking.

These instruments allow the acquisition of HSI images in reflectance mode, using IQ studio software (version 2021, Specim, Spectral Imaging Ltd., Oulu, Finland) for VisNIR camera, and Lumo-Scanner software (version 2022, Lumo-Scanner, Specim, Spectral Imaging Ltd., Oulu, Finland) for the SWIR system. The parameters of the VisNIR instrument were set as follows: spatial resolution: 512 × 512 pixels; spectral resolution: 7 nm; integration time: 3.0 ms. The parameters of the SWIR instrument were set as follows: spectral resolution: 8 nm; spatial resolution: 640 pixels; exposure time: 4.70 ms; frame rate: 15.20 Hz; positioning speed of the platform: 20.00 mm s^−1^; scanning speed to 5.84 mm s^−1^. The reflectance of each image was corrected using a calibrated white and dark reference, according to the following equation:

$$I=\frac{I_{raw}-I_{B}}{I_{W}-I_{B}}$$

where I = the corrected reflectance, I_raw_ = the original reflectance, I_B_ = the black reference, and I_W_ = the white reference.

**Figure 1:**
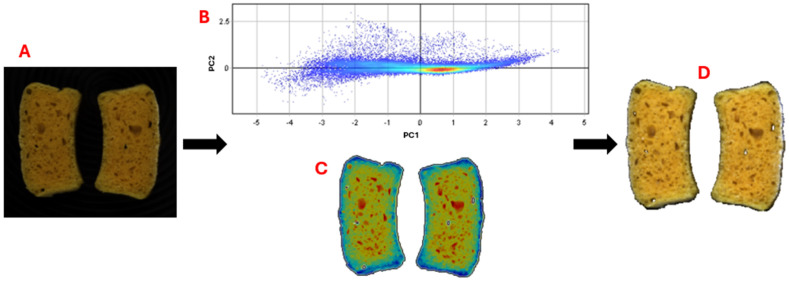
Example image of bread slices (**A**) before processing; PCA score plot of pixels (**B**); PCA score image (**C**); RGB image after processing (**D**).

The processing of HSI images was carried out using Evince software (version 2.7.12, Prediktera AB, Umea, Sweden). The processing of the HSI images consisted of three steps (Figure 1). The first step regards the image segmentation, for the removal of the background and the extraction of the pixels of each sample from the whole hyperspectral image, using principal component analysis (PCA). The second step consists of a baseline correction and a smoothing with the application of the first order Savitzky–Golay derivative (second-degree polynomial and seven-point window). In the third step, mean spectrum is obtained as average of the spectra related to all pixels for each sample, considering the overall hyperspectral image. Finally, first-derivative reflectance spectra were used to highlight chemical differences during the storage time.

### 2.11. Statistical Analysis

The results of all parameters were expressed as mean ± standard deviations (SD) for three replications. Analysis of variance (Kruskal–Wallis test) and the post hoc non-parametric Dunns’ range test were performed, with the level of significance set at *p* ≤ 0.05.

Principal component analysis (PCA) was carried out on spectral data for each type of bread and for each time to verify if the hyperspectral imaging device was able to recognize the presence of mold on bread. PCA was processed using the SPSS statistical software (Version 22.0; IBM Corp., Armonk, NY, USA).

## 3. Results and Discussion

### 3.1. Raw Material Characterization

#### 3.1.1. Proximate Composition

Table 3 shows the biochemical composition of sorghum byproducts. Stalk samples showed high values of moisture, whereas values of debranning products (C3 and C4) were quite similar. The highest protein content was for C4 and the lowest for C1. The stalk samples were shown to be rich in minerals, in contrast to the bran fractions (Table 3). Stalk samples exhibited double the fiber content of debranning products. The results of the stalks were in accordance with Singh et al. [28], who analyzed the variation in carbohydrate and protein fractions in stover of sorghum cultivars. Regarding debranning products, Miafo et al. [29] reported that sorghum bran consists of 10 to 25% of the total whole grain and it is rich in dietary fiber (36 to 50% of total dietary fiber and 35 to 48% of insoluble dietary fiber). Our results are slightly lower. Moreover, sorghum bran can be a source of soluble dietary fiber with antioxidant properties, such as glucoarabinoxylans, as highlighted by Ayala-Soto et al. [30]. However, proximate composition of stalks and debranning products can be influenced by several factors like variety, agronomic management, growth stage, and environmental conditions during growth [31,32]. In fact, slight but significant differences in proximal composition were observed between samples from the two different management (organic C1–C4 and integrated C2–C3), except for TDF in stalk samples.

The wheat flour proximal composition showed a moisture content of 8.8 g 100 g^−1^ DW, a protein content of 13.2 g 100 g^−1^ DW, an ash content of 3.92 g 100 g^−1^ DW, and a total dietary fiber content of 2.0 g 100 g^−1^ DW.

#### 3.1.2. Characterization with HSI

**Figure 2:**
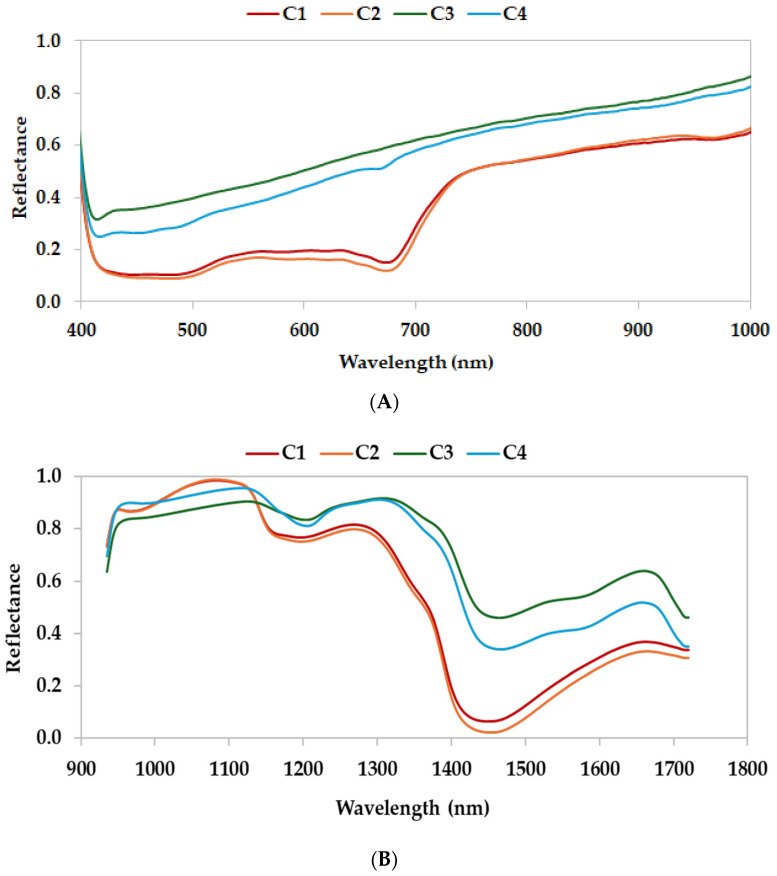
(**A**) VisNIR spectral profiles of different sorghums samples. (**B**) SWIR spectral profiles of different sorghum samples. Legend: C1 = stalk from organic management; C2 = stalk from integrated management; C3 = debranning products from integrated management; C4 = debranning products from organic management.

Figure 2 depicts the average reflectance curves of the four samples of sorghum byproducts in the visible-light and near-infrared spectral regions. Curves of stalk samples and those of debranning product samples showed similar spectral profiles with slight differences. As reported by Huang et al. [33], the spectra reflected the chemical composition of the main components in the sorghum samples. In the visible range (400–780 nm), the average reflectance curves of C1 and C2 showed interesting peaks around 550 nm and 750 nm, whereas the spectra of C3–C4 displayed no obvious absorption peaks (Figure 2A). Spectra in the visible region mainly reflected the color information of the samples. The color characteristics of the stalks were strongly affected by several pigments, such as chlorophylls, whereas debranning products were characterized by different phenolic compounds, such as gallic acid and proanthocyanidins, and yellow pigment that could have contributed to the different absorption peak. The near-infrared region (800–1000 nm) revealed high absorption that could be assigned to third overtones of O–H and C–H, mainly related to carbohydrate and water content [34,35,36]. As illustrated in Figure 2B, six notable absorption peaks appeared at around 920, 980, 1020, 1130, 1480, and 1680 nm. The peaks at about 920 and 1480 nm correspond to the second and first vibrational overtones of the O–H bond, respectively, and their intensity variations primarily reflect differences in moisture content among the sorghum samples [34]. The absorption at around 980 nm can be attributed to the second overtone of O–H bond stretching and mainly associated with the presence of carbohydrates in sorghum [35]. The absorption feature at around 1020 nm can be primarily related to the second overtone of C–H bond stretching [36]. Similarly, the peak observed at around 1130 nm is originated from C–H stretching overtones and it is mainly linked to the carbohydrates and fats contents [37]. Absorption at around 1200–1300 nm is largely due to the second overtone stretching of C–H bonds, reflecting the presence of carbohydrates and lipids in sorghum [38]. The peak at 1480 nm is influenced by N–H bond absorption, which is typically associated with protein content [39]. The spectral profiles of various sorghum samples can exhibit significant differences across certain wavelength regions. By examining these spectral variations with the molecular bonds and functional groups corresponding to each absorption feature, it is possible to infer differences in the levels of water, carbohydrates, fats, and proteins among sorghum tissues, as previously reported by Hu et al. [40].

### 3.2. Optimization of UAE Process Parameters and Validation of the RSM Models

Based on the BBD, experimental tests (runs) were carried out to extract TPC by UAE. The results of the 15 tests for each of the 4 samples are reported in Table 4. The results of runs 13, 14, and 15 are equivalent, as expected from BBD.

With regard to the C1 sample (stalks from organic farming), the lowest mean value of TPC was recorded for run 9 (solvent composition = 65% ethanol/H_2_O (*v/v*), extraction time = 40 min, extraction temperature = 70 °C), while the highest mean value was for run 11 (solvent composition = 65% ethanol/H_2_O (*v/v*), extraction time = 40 min, extraction temperature = 80 °C). Regarding the C2 sample (stalks from integrated farming), the lowest mean TPC value was for run 2 (solvent composition = 80% ethanol/H_2_O (*v/v*), extraction time = 40 min, extraction temperature = 75 °C), while the highest mean value was for run 12 (solvent composition = 65% ethanol/H_2_O (*v/v*), extraction time = 60 min, extraction temperature = 80 °C). With regard to C3 sample (debranning products from organic farming), the lowest mean TPC value was recorded for runs 13–15 (solvent composition = 65% ethanol/H_2_O (*v/v*), extraction time = 50 min, extraction temperature = 75 °C), while the highest mean value was recorded for run 2 (solvent composition = 80% ethanol/H_2_O (*v/v*), extraction time = 40 min, extraction temperature = 75 °C). Regarding the C4 sample (debranning products from integrated farming), the lowest mean TPC value was for run 9 (solvent composition = 65% ethanol/H_2_O (*v/v*), extraction time = 40 min, extraction temperature = 70 °C), while the highest mean value was for run 2 (solvent composition = 80% ethanol/H_2_O (*v/v*), extraction time = 40 min, extraction temperature = 75 °C).

The regression models for the predicted response (TPC_RSM_) for the four samples were the following:

$${TPC}_{RSM\_C1}=-1870.99-2.38X_{1}+20.95X_{2}+40.40X_{3}-0.01X_{1}^{2}-0.17X_{3}^{2}-0.03X_{1}X_{2}+0.07X_{1}X_{3}-0.26X_{2}X_{3}$$

$${TPC}_{RSM\_C2}=1036.34+15.84X_{1}-2.11X_{2}-34.27X_{3}-0.05X_{1}^{2}-0.29X_{3}^{2}+0.02X_{1}X_{2}-0.15X_{1}X_{3}-0.02X_{2}X_{3}$$

$${TPC}_{RSM\_C3}=-4.88-10.63X_{1}-3.76X_{2}+16.44X_{3}+0.08X_{1}^{2}+0.04X_{2}^{2}-0.11X_{3}^{2}-0.01X_{1}X_{2}+0.004X_{1}X_{3}+0.005X_{2}X_{3}$$

$${TPC}_{RSM\_C4}=5322.14-10.15X_{1}-17.04X_{2}-115.66X_{3}+0.09X_{1}^{2}+0.30X_{2}^{2}+0.78X_{3}^{2}-0.08X_{1}X_{2}+0.05X_{1}X_{3}-0.10X_{2}X_{3}$$

where X_1_, X_2_, and X_3_ are the variables for green solvent composition, extraction time, and extraction temperature, respectively.

Table 5 exhibits the analysis of variance for the second-order polynomial equation for ultrasound-assisted extraction of total phenolic compounds, to evaluate the models’ adequacy. The linear, quadratic, and two-factor interaction effect coefficients, the statistical significance (*p*-value), the coefficient of determination R^2^, the adjusted coefficient of determination R_adj_^2^, and the lack of fit value were shown. All the models were highly significant (*p* < 0.0001), describing a high degree of correlation between the experimental and predicted values. In fact, all R^2^ and R_adj_^2^ were higher than 0.95. Moreover, all *p*-values of lack of fit were not significant. Therefore, the models can be accepted.

Experimental tests (runs) were carried out also for AA (Table 6). AA of sample C1 (stalks from organic farming) showed the lowest mean value at run 4 (solvent composition = 80% ethanol/H_2_O (*v*/*v*), extraction time = 60 min, extraction temperature = 75 °C), while the highest mean value at run 5 (solvent composition = 50% ethanol/H_2_O (*v/v*), extraction time = 50 min, extraction temperature = 70 °C) (Table 6). With regard to the C2 sample (stalks from integrated farming), the lowest mean value of antioxidant activity was obtained for run 6 (solvent composition = 80% ethanol/H_2_O (*v/v*), extraction time = 50 min, extraction temperature = 70 °C), while the highest mean value was carried out for run 11 (solvent composition = 65% ethanol/H_2_O (*v/v*), extraction time = 40 min, extraction temperature = 80 °C). Regarding C3 sample (debranning products from organic farming), the lowest mean value of AA was obtained for run 12 (solvent composition = 65% ethanol/H_2_O (*v/v*), extraction time = 60 min, extraction temperature = 80 °C), while the highest mean value for runs 13–15 (solvent composition = 65% ethanol/H_2_O (*v/v*), extraction time = 50 min, extraction temperature = 75 °C). With regard to the sample C4 (debranning products from integrated farming), the lowest mean value of AA was recorded for run 3 (solvent composition = 50% ethanol/H_2_O (*v/v*), extraction time = 60 min, extraction temperature = 75 °C), while the highest mean value for run 9 (solvent composition = 65% ethanol/H_2_O (*v/v*), extraction time = 40 min, extraction temperature = 70 °C).

The regression models for the predicted response (AA_RSM_) for the four samples were as follows:

$${AA}_{RSM\_C1}=168.01+0.95X_{1}+2.09X_{2}-5.89X_{3}+0.002X_{1}^{2}-0.02X_{2}^{2}+0.04X_{3}^{2}-0.01X_{1}X_{2}-0.01X_{1}X_{3}+0.01X_{2}X_{3}$$

$${AA}_{RSM\_C2}=360.46-2.12X_{1}+0.56X_{2}-7.48X_{3}-0.002X_{1}^{2}+0.01X_{2}^{2}+0.05X_{3}^{2}+0.01X_{1}X_{2}+0.02X_{1}X_{3}-0.03X_{2}X_{3}$$

$${AA}_{RSM\_C3}=148.74+2.65X_{1}+0.01X_{2}-4.78X_{3}-0.02X_{1}^{2}+0.01X_{2}^{2}+0.03X_{3}^{2}+0.001X_{1}X_{2}-0.01X_{1}X_{3}-0.001X_{2}X_{3}$$

$${AA}_{RSM\_C4}=-495.65-2.14X_{1}+3.86X_{2}+13.39X_{3}-0.003X_{1}^{2}-0.02X_{2}^{2}-0.10X_{3}^{2}+0.002X_{1}X_{2}+0.03X_{1}X_{3}-0.03X_{2}X_{3}$$

where X_1_, X_2_, and X_3_ are the variables for green solvent composition, extraction time, and extraction temperature, respectively.

ANOVA for AA (Table 7) showed high coefficient of determination R^2^ (>0.93) and adjusted coefficient of determination R_adj_^2^ (>0.90) for all models, indicating a good agreement between the predicted and measured values.

**Figure 3:**
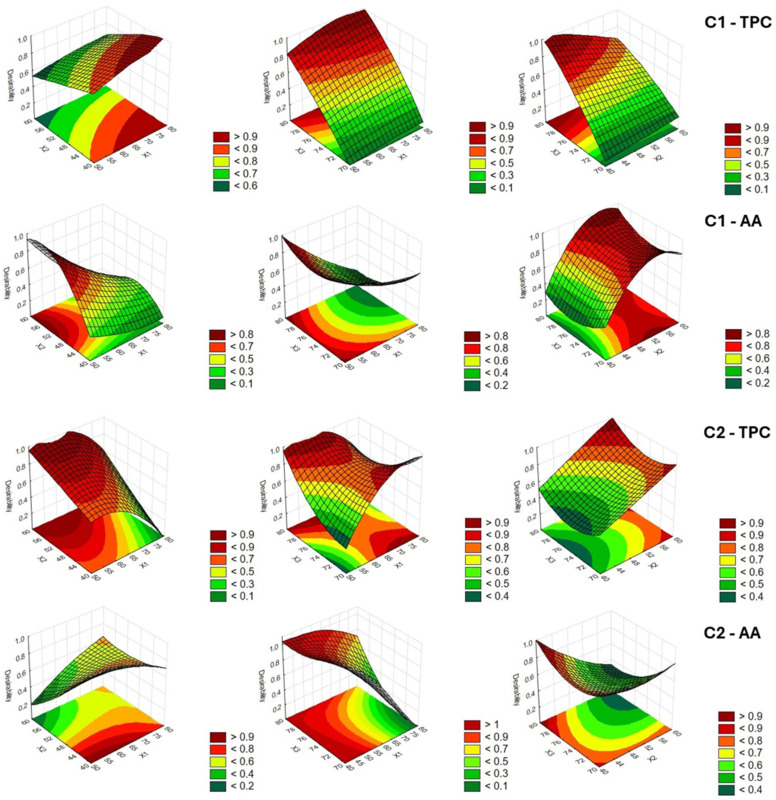
Response surface and contour plots of extracts of C1 and C2 samples for total phenolic compounds (TPC) and antioxidant activity (AA). Legend: C1 = stalk from organic management; C2 = stalk from integrated management.

**Figure 4:**
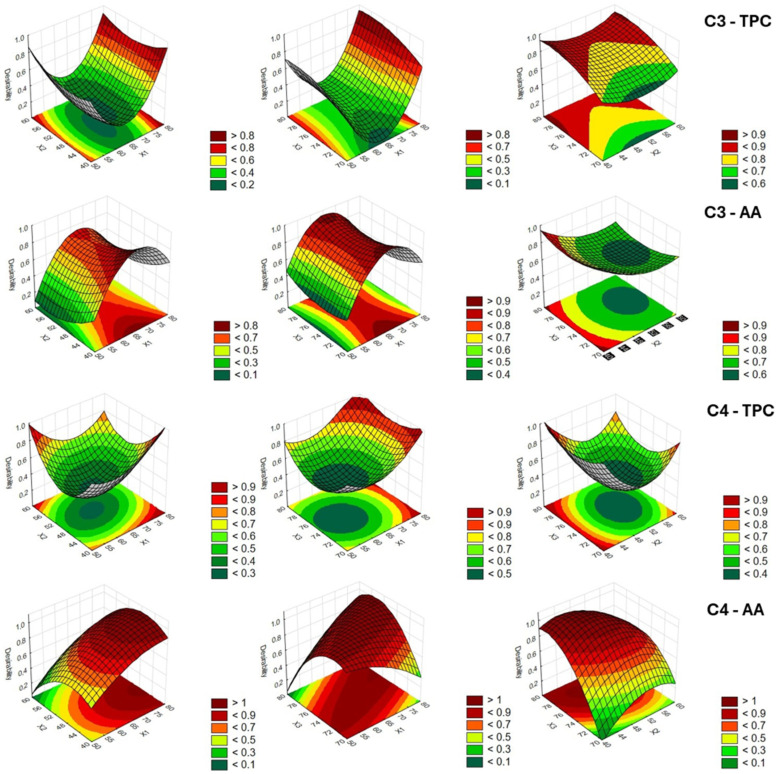
Response surface and contour plots of extracts of C3 and C4 samples for total phenolic compounds (TPC) and antioxidant activity (AA). Legend: C3 = debranning products from integrated management; C4 = debranning products from organic management.

Three-dimensional response surface plots were used to assess the effects of different combinations of levels of each pair of independent variables (X_1_–X_3_) on the overall response desirability (Figure 3 and Figure 4) for TPC and AA. They can help visualize the interactions between variables. The desirability function ranges from 0.0 (undesirable) up to 1.0 (very desirable). Therefore, highly desirable values for TPC and AA are depicted in red for each pair of process variables. Simultaneous comparison of the desirability functions allowed estimation of the optimal extraction values for green solvent composition, extraction time, and extraction temperature, as reported in Table 8. The optimal conditions were determined by maximizing the response functions.

The comparison related to the two variables (TPC and AA) and the four samples can provide some general indications. The analysis of the models highlighted the possibility of obtaining an extract richer in phenols with a high ethanol content in solvent composition. This may be due to the fact that phenolic compounds are more soluble in an ethanol/water solution than in a pure solvent [16]. The extraction yield in TPC could increase due to the action of water that promotes the swelling of the cellular material, and therefore increases surface contact between plant matrix and solvent, with a consequent improvement of cell wall permeability [41,42]. At the same time, high temperatures can favor the extraction, increasing the release rate of the phytocompounds in the solvent, also taking into account the greater dissociation of the polyphenols bound to the membrane [43]. Moreover, time reduction is suggested for the preservation of thermolabile and/or unstable polyphenols, as well as for savings in the cost per unit volume of the extract [44,45]. Luo et al. [46] used UAE to extract TPC from red sorghum (*Sorghum bicolor* L.) bran. Although the authors considered different raw materials and different process factors and levels, some considerations may be common to the two studies. In fact, they observed that the yield of TPC increased as the ultrasonic time ascended, and an ethanol concentration of 30–70% was good for extracting TPC, with the best concentration at 50%. The importance of ethanol in the extraction of phenolic compounds was also highlighted by Barros et al. [47]. They used an accelerated solvent extractor (ASE) to extract polyphenols from black and tannin sorghum bran. The ASE at temperatures above 100 °C using water and ethanol/water (50% and 70%) solvent significantly improved the extraction of phenolic compounds from black sorghum bran compared to conventional extractions using the same solvents. Furthermore, the solvent at 70% ethanol: water ratio carried out the highest yield in TPC.

AA showed its best performance at moderate times and low ethanol concentration, except for C4, and lower temperatures, especially for debranning products. Temperature can amplify solubility and diffusion through increased molecular motion while preventing degradation [42]. More concentrated ethanol can also extract non-antioxidant or pro-oxidant compounds, which dilute the antioxidant effect of the overall extracted pool [48]. Antioxidant compounds, especially polyphenols, are sensitive to oxidation. Longer extraction times expose them to oxygen, ultrasound, and heat for longer, increasing the risk of degradation. In addition, ultrasonic energy applied for long periods can generate free radicals (such as OH• radicals from water), which degrade antioxidant compounds.

Under the optimal conditions (Table 8), the extractions were repeated and compared to those obtained with the traditional method (Table 9).

All measurements showed higher TPC, TFC, and pigment contents and higher AA values with UAE in comparison with the traditional technique. Many studies confirmed the validity of the UAE extraction in obtaining a higher yield of phenols, pigment, and a corresponding antioxidant activity [16,44,49,50,51]. This is mainly due to the increase in the solute solubility, the favoring of cell disruption, the enhancing of mass transfer, and the improvement in solvent penetration [52,53,54].

### 3.3. Characterization of Phenolic Acids Extract by HPLC-DAD

HPLC chromatograms were used to investigate sorghum phenolic acids compositions among sorghum samples extracted with different ethanol: water ratios. In accordance with the retention time of commercially available phenolic standards, PAs were identified in sorghum extracts, most of which were more prominent under the detection wavelengths of 280 and 350 nm. The concentrations of phenolics in different extracting solvent procedures (ultrasound: UAE; traditional: TM) are shown in Table 10. The content of PAs significantly varied (*p* ≤ 0.05) between different extraction systems and among sorghum samples. Many studies reported a different HPLC phenolic compounds profile when different extraction systems, sorghum genotypes, or histological tissues were studied [55,56,57,58].

All extracts with ethanol obtained from UAE procedures showed higher concentrations of PAs than those obtained from the classic extraction technique, except for vanillic acid in C4 and 4-coumaric acid in C3 and C4. A similar trend was reported by Hong et al. [55]. Generally, phenolics tend to be extracted in greater amounts using more polar solvents and applying physical treatment, such as microwave and ultrasounds. Differences in phenols profiles can strongly affect the potential biological activities of the extracts, including antimicrobial, antiproliferative, and proapoptotic effects against colon cancer cells [59]. In particular, Denardi-Souza et al. [59] reported a significant antimicrobial action of specific phenolic acids, such as gallic and ferulic acids, against different microorganisms, increasing bread shelf life.

### 3.4. Effect of Sorghum Stalk Optimal Extract on Bread

Crumb and crust color characteristics for the three types of bread are shown in Table 11. Regarding the crust color, the enrichment with the extract significantly affected only the coordinate b*: the yellowness was higher for P2. On the contrary, significant differences in all CIELab coordinates were observed among the bread crumbs. The luminosity, the redness, and the yellowness of the bread increased as the amount of extract increased. Color variation could be ascribed to the chemical composition of C1 extract (Table 10) used to enrich the dough, characterized by high amount of phenols, chlorophyll, and yellow pigment [60,61]. In particular, the red shift for enriched breads may be due to the presence of gallic acid in the extract, as emerged from chromatographic analysis. The increase in yellowness and luminosity may be ascribed to flavonoids, which are abundant in extracts from sorghum obtained with high amount of ethanol in solvent composition [55].

Processing and cooking can affect the concentrations of bioactive compounds in the final products, because these compounds can be thermolabile [24,62]. Therefore, TPC and AA were evaluated in breads to verify whether the different concentrations of the C1 extract brought about a significant increase in TPC and AA in P2 and P3 compared to the control bread (P1). Table 12 shows significant differences for both variables. The 10% enrichment produced an increase of 15.3% of TPC and 12.8% of AA. The effect of the extract on bread enriched at 20% was much more accentuated: increases of 12.8% of TPC and 79.5% of AA was observed. A similar trend was also observed by Chen et al. [63], who reported an increase in phenolic compounds and antioxidant activity in bread enriched with sorghum bran extract.

One of the major problems that arise during the storage of bread is the appearance of mold, as it causes economic losses and dissatisfaction among consumers [64]. This is strictly related to the type of bread, the method, and the temperature of storage [65]. Various molds can appear in wheat bread, such as Penicillium, Aspergillus, Cladosporium, Mucorales and Neurospora [65,66,67].

**Figure 5:**
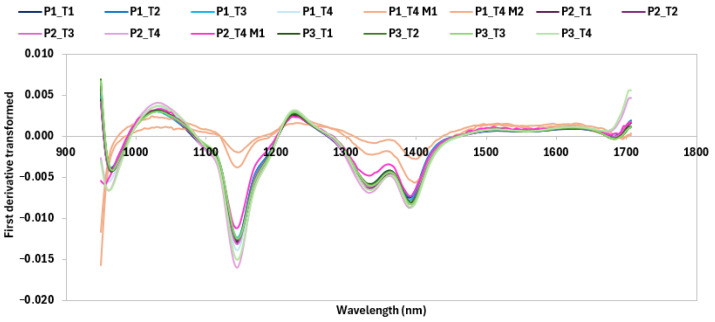
Mean spectra first derivative of each bread (P1 = control; P2 = bread with 10% of extract; P3 = bread with 20% of extract) at different storage days (T1 = second day after cooking; T2 = third day after cooking; T3 = fourth day after cooking; T4 = seventh day after cooking). The suffix M indicates the presence of mold.

Considering that sorghum byproducts showed a high amount of bioactive compounds, we verified if and to what extent the extract with the highest TPC content (C1) could influence the bread shelf life. Therefore, three types of bread were produced: a control (P1), a bread enriched with 10% extract (P2), and a bread enriched with 20% extract (P3). Bread samples were stored in sealed bags. Every day, hyperspectral image acquisitions were carried out with the SWIR camera, until the appearance of mold (Figure 5).

Significant differences were observed among the spectra of bread samples at different storage times. In particular, a high variation of spectra reflectance was observed at about 1150 and 1350 nm when mold appeared. A similar variation of SWIR signals was observed in wheat kernel and flour after fusarium infection by Alisaac et al. [68].

**Figure 6:**
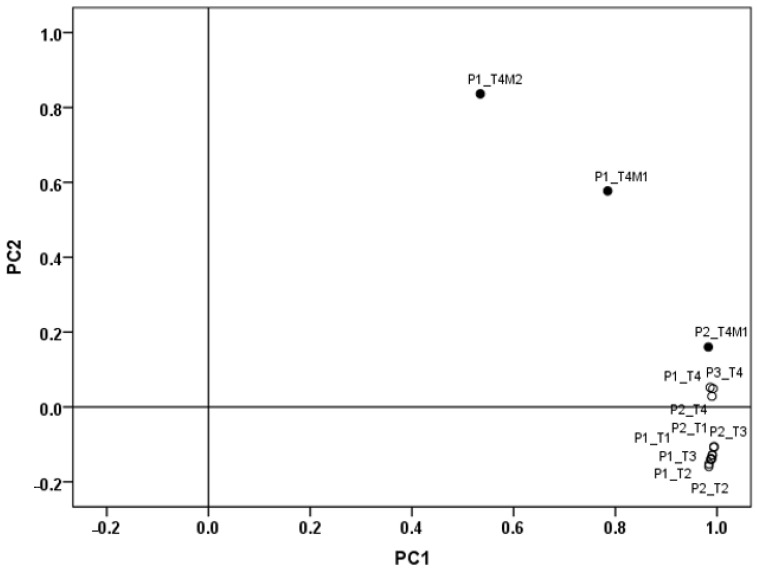
Score plot from principal component analysis performed on mean spectra of each bread (P1 = control; P2 = bread with 10% of extract; P3 = bread with 20% of extract) for 4 times (T1 = second day after cooking; T2 = third day after cooking; T3 = fourth day after cooking; T4 = seventh day after cooking). The suffix M and the solid circle indicate the presence of mold.

The mean spectra for each type of bread were calculated for four times, for which a principal component analysis was performed to verify the camera’s ability to identify the appearance of mold, based on the change in the chemical composition of the spectra. A score plot from principal component analysis performed on the mean spectra of each bread for four times (Figure 6) showed the similarities among all different types of bread during storage at room temperature (about 19 °C). The first two principal components accounted cumulatively for 98.9% of the explained variance: PC1 explained 90.7% and PC2 8.2% of the total variance. The score plot highlighted a discrimination between the spectra of the breads at times T1–T3 and those at T4, and especially those relating to the areas affected by mold in P1 and P2 breads. The separation was clearer for the control bread as the presence of mold at time T4 was extremely evident, compared to the P2 bread, which presented barely visible mold. In general, bread shelf life and spoilage depend on its moisture content [69]. However, in our study, all breads were made with the same addition of liquids part. Therefore, the behavior of P3 could be due to the addition of extract.

These results confirmed that the natural extract with high amount of bioactive compounds could positively influence the maintenance of the bread product, and the ability of the HSI technique to conduct early inspection and monitor bread spoilage. Several studies attributed to the phenolic compounds an antimicrobial activity [70,71,72]. In particular, Borges et al. [73] and Chen et al. [74] ascribed to the phenolic acids, such as gallic and ferulic acids, the ability to destroy the integrity of microbial cell membranes, resulting in the leakage of intracellular substances. Furthermore, flavonoids can inhibit energy metabolism in cells and inhibit essential enzymes required for DNA and RNA replication, resulting in cell death [75].

Although there are many previous studies that have developed prediction models of food quality parameters using HSI data storage [76], few studies have investigated the potential of spectroscopic techniques to assess microbiological deterioration and shelf life of baked goods post-baking. Saleem et al. examined the microbial spoilage growth of sponge cake using a VisNIR hyperspectral camera (395–1000 nm); the method successfully allowed them to train the model to distinguish between healthy and spoiled samples with 98% accuracy [77]. Cevoli et al. used NIR spectroscopy coupled with principal component analysis to monitor the shelf life of a traditional Italian flat bread Piadina, improved with sourdough fermentation and enzymes, and to predict storage time [78]. Mehdizadeh et al. explored the ability of the VisNIR hyperspectral camera (400–950 nm) to evaluate bread texture over six days of storage, highlighting changes in crumb moisture, firmness, chewiness, cohesiveness, and springiness [79].

### 3.5. Sensorial Analysis

**Figure 7:**
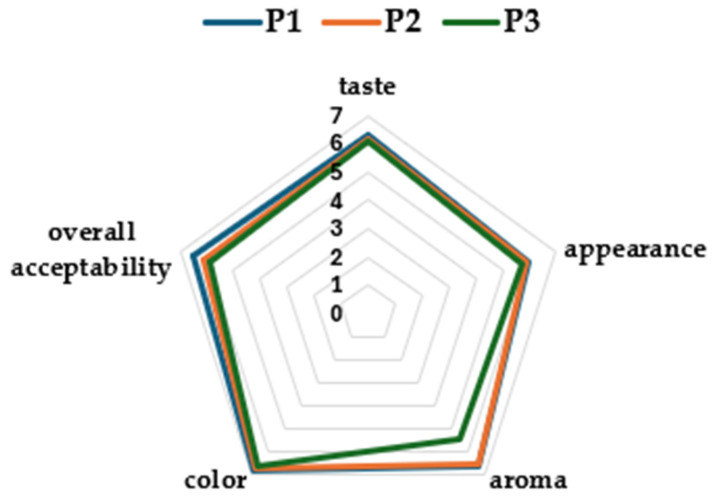
Sensory analysis of bread with different extracts enrichment (P1 = control; P2 = bread with 10% of extract; P3 = bread with 20% of extract).

The mean intensity scores for organoleptic attributes for each type of bread are shown in Figure 7. The consumer test revealed the bread samples most preferred by consumers in terms of taste, color, aroma, appearance, and overall preference. No significant differences (*p* < 0.05) among breads were found for the overall acceptability and the sensorial attributes, except for aroma. The score of this attribute was significantly lower in P3 than those of the other two breads. This may be related to the grassy smell of the sorghum stalk extract.

Sensory analysis of bread, including its smell, is a crucial aspect of evaluating its quality and consumer appeal. The grass smell, which is perceived by consumers as a negative aspect, could be related to the presence of specific and not desired volatile compounds in the stalk extract. In the context of bread, the grass smell can be associated with a “green”, “grass-like”, or “vegetative” note, though less common than other bread aroma compounds (like those from yeast, sugars, and flour). Karl et al. analyzed samples of sorghum stems with leaves with online proton transfer reaction mass spectrometry and proton transfer ion trap mass spectrometry methods [80]. These samples released a large variety of oxygenated volatile organic compounds, including methanol, acetaldehyde, acetone, n-pentanal, methyl propanal, hexenol, hexanal, cis-3-hexenal, and trans-2-hexenal, which could be responsible for the lower score of aroma for P3 bread.

## 4. Conclusions

In the current agricultural and food industry, a pressing challenge is the reduction in waste production and the promotion of its reuse through the application of green technologies, such as UAE coupling with RSM and use of green solvents. Through RSM, we identified different optimal extraction conditions based on the type of residues (stalks or debranning products). Under optimal conditions, UAE resulted in over 80% higher phenolic yield and more than 25% greater antioxidant activity compared to conventional extraction methods. HPLC analysis revealed noteworthy phenolic acid profiles, with high levels of ferulic and gallic acids in stalks samples, and caffeic and 4-coumaric acids in debranning products. These compounds are known for their antioxidant and antimicrobial properties and could be used to obtain food with increased potential nutritional and prolonged shelf life.

Significant enhancements in total phenolic compounds and antioxidant activity were observed in bread enriched with the optimized extract from samples showing the maximum phenolic amount, along with an extension in shelf life.

Our findings highlighted that fine-tuning the physical parameters of the UAE process enhances the recovery of bioactive phenolic compounds, which are key to biological functionality. Consequently, these extracts show promise as additives or ingredients for natural, health-promoting products in food, pharmaceutical, and cosmetic industries. Nonetheless, further research is needed to explore the full range of eco-friendly extract applications. Moreover, transferring these applications at scale can involve further research to ensure the maximization of extraction efficiency and reproducibility.

## Figures and Tables

**Table 1:** Samples of sorghum byproducts used in this study.

Sample	Type of Waste	Type of Farm
C1	Stalk	Organic
C2	Stalk	Integrated
C3	Debranning products	Integrated
C4	Debranning products	Organic

**Table 2:** Box–Behnken design with coded and uncoded parameters of ultrasound-assisted extraction (UAE).

Run	X_1_	X_2_	X_3_	X_1_ (%)	X_2_ (min)	X_3_ (°C)
1	−1	−1	0	50	40	75
2	1	−1	0	80	40	75
3	−1	1	0	50	60	75
4	1	1	0	80	60	75
5	−1	0	−1	50	50	70
6	1	0	−1	80	50	70
7	−1	0	1	50	50	80
8	1	0	1	80	50	80
9	0	−1	−1	65	40	70
10	0	1	−1	65	60	70
11	0	−1	1	65	40	80
12	0	1	1	65	60	80
13	0	0	0	65	50	75
14	0	0	0	65	50	75
15	0	0	0	65	50	75

Legend: X_1_ = green solvent composition (%); X_2_ = extraction time (min); X_3_ = extraction temperature (°C).

**Table 3:** Proximate composition (moisture, protein, ash, and total dietary fiber (TDF) of sorghum byproducts.

Sample	Moisture (g 100 g^−1^ FW)	Protein (g 100 g^−1^ DW)	Ash (g 100 g^−1^ DW)	TDF (g 100 g^−1^ DW)
C1	63.1 ± 0.5 ^b^	8.1 ± 0.2 ^b^	8.48 ± 0.03 ^b^	60.4 ± 1.4 ^a^
C2	69.6 ± 0.8 ^a^	9.9 ± 0.2 ^a^	9.03 ± 0.01 ^a^	61.7 ± 0.3 ^a^
C3	11.4 ± 0.3 ^c^	10.5 ± 0.1 ^d^	2.64 ± 0.01 ^d^	32.1 ± 0.7 ^b^
C4	11.7 ± 0.3 ^c^	13.5 ± 0.1 ^c^	4.00 ± 0.02 ^c^	29.8 ± 0.4 ^c^

Legend: FW = fresh weight; DW = dry weight; C1 = stalk from organic management; C2 = stalk from integrated management; C3 = debranning products from integrated management; C4 = debranning products from organic management; Results are expressed as mean ± standard deviation for three replications. Values with different letters in the same parameter are significantly different (*p* ≤ 0.05).

**Table 4:** Experimental and predicted response values (total phenolic compounds, TPC, in mg GAE 100 g^−1^ FW) in the extracts using response surface methodology (RSM).

	C1		C2		C3		C4	
Run	TPC_exp_	TPC_RSM_	TPC_exp_	TPC_RSM_	TPC_exp_	TPC_RSM_	TPC_exp_	TPC_RSM_
1	326 ± 2	324	234 ± 1	234	226 ± 3	227	257 ± 1	256
2	340 ± 4	343	228 ± 1	229	234 ± 4	233	288 ± 4	285
3	326 ± 1	323	248 ± 3	247	230 ± 4	231	278 ± 1	281
4	323 ± 6	324	256 ± 3	255	233 ± 1	232	259 ± 4	259
5	291 ± 6	292	235 ± 1	235	219 ± 5	218	265 ± 1	263
6	294 ± 3	292	256 ± 1	259	221 ± 4	222	260 ± 2	260
7	344 ± 2	346	264 ± 1	260	227 ± 2	226	253 ± 1	253
8	368 ± 5	366	241 ± 2	240	229 ± 1	230	262 ± 1	264
9	286 ± 8	286	250 ± 3	249	205 ± 1	205	265 ± 2	268
10	302 ± 1	302	267 ± 1	266	206 ± 3	206	277 ± 6	277
11	377 ± 2	376	249 ± 1	250	212 ± 1	212	275 ± 2	275
12	340 ± 3	340	270 ± 2	271	214 ± 2	214	267 ± 2	264
13	330 ± 1	330	252 ± 4	252	208 ± 1	208	221 ± 1	221
14	330 ± 1	330	252 ± 4	252	208 ± 1	208	221 ± 1	221
15	330 ± 1	330	252 ± 4	252	208 ± 1	208	221 ± 1	221

Legend: C1 = stalk from organic management; C2 = stalk from integrated management; C3 = debranning products from integrated management; C4 = debranning products from organic management; exp = experimental; RSM = response surface methodology. Results are expressed as mean ± standard deviation for three replications.

**Table 5:** Analysis of variance for the second-order polynomial equation for ultrasound-assisted extraction of total phenolic compounds.

	C1		C2		C3		C4	
	Sum of Square	*p*-Value	Sum of Square	*p*-Value	Sum of Square	*p*-Value	Sum of Square	*p*-Value
X_1_	370.01	<0.0001	0.20	0.8614	63.37	0.0015	57.20	0.0138
X_2_	369.48	0.1711	1529.25	<0.0001	11.98	0.1261	2.88	0.5518
X_3_	16,562.61	<0.0001	59.53	0.0064	242.57	<0.0001	34.26	0.0497
X_1_^2^	25.31	0.8805	770.88	<0.0001	2659.80	<0.0001	2692.59	<0.0001
X_2_^2^	0.29	<0.0001	0.01	0.9776	125.43	<0.0001	6711.89	<0.0001
X_3_^2^	136.81	0.0035	381.20	<0.0001	53.27	0.0031	2828.28	<0.0001
X_1_ X_2_	144.05	0.0028	88.13	0.0014	16.05	0.0794	1299.16	<0.0001
X_1_ X_3_	213.18	0.0005	976.22	<0.0001	0.58	0.7290	97.75	0.0021
X_2_ X_3_	1392.16	<0.0001	10.02	0.2262	0.40	0.7728	207.11	<0.0001
Residual	264.66		128.50		93.99		136.98	
Lack of fit	69.57	0.1308	39.35	0.0941	11.79	0.5044	55.02	0.0999
Pure error	195.09		89.15		82.21		81.96	
Total SS	19,475.60		3943.94		3281.72		12,582.01	
R^2^	0.986		0.968		0971		0.9875	
R_adj_^2^	0.981		0.954		0.958		0.9819	
AAD								

Legend: X_1_ = green solvent composition; X_2_ = extraction time; X_3_ = extraction temperature; Legend: C1 = stalk from organic management; C2 = stalk from integrated management; C3 = debranning products from integrated management; C4 = debranning products from organic management.

**Table 6:** Experimental and predicted response values (antioxidant activity, AA, in mg TE 100 g^−1^ FW) in the extracts using response surface methodology (RSM).

	C1		C2		C3		C4	
Run	AA_exp_	AA_RSM_	AA_exp_	AA_RSM_	AA_exp_	AA_RSM_	AA_exp_	AA_RSM_
1	20.2 ± 0.1	20.4	26.2 ± 0.4	26.4	20.9 ± 1.3	21.0	27.9 ± 0.1	28.0
2	20.7 ± 0.4	20.6	20.1 ± 0.1	20.1	22.3 ± 1.0	22.5	28.8 ± 0.7	28.7
3	23.9 ± 0.1	24.1	22.0 ± 0.8	22.1	18.6 ± 0.5	18.5	26.6 ± 0.5	26.7
4	19.1 ± 0.1	19.0	22.3 ± 0.3	22.2	20.7 ± 0.8	20.6	28.5 ± 0.9	28.4
5	25.6 ± 0.3	25.3	26.5 ± 0.4	26.2	19.2 ± 0.9	19.2	29.6 ± 0.4	29.7
6	24.5 ± 0.3	24.5	19.7 ± 0.7	19.7	21.9 ± 1.4	21.9	25.6 ± 0.2	25.9
7	25.2 ± 0.9	25.2	23.4 ± 0.3	23.4	19.7 ± 1.7	19.8	24.1 ± 0.9	23.9
8	20.8 ± 0.7	21.1	23.5 ± 0.1	23.7	20.8 ± 0.3	20.8	30.2 ± 0.7	30.1
9	22.1 ± 0.7	22.3	23.2 ± 0.2	23.3	26.7 ± 0.2	26.6	25.9 ± 0.9	25.7
10	22.4 ± 0.3	22.6	24.5 ± 0.4	24.7	24.3 ± 1.0	24.4	28.0 ± 0.4	27.8
11	20.0 ± 0.4	19.9	26.6 ± 0.3	26.4	26.5 ± 0.5	26.4	27.7 ± 0.2	27.9
12	21.8 ± 0.1	21.6	22.8 ± 0.1	22.7	24.0 ± 0.8	24.1	23.8 ± 0.4	24.1
13	22.6 ± 0.3	22.6	22.4 ± 0.6	22.4	23.4 ± 0.2	23.4	30.5 ± 0.2	30.5
14	22.6 ± 0.3	22.6	22.4 ± 0.6	22.4	23.4 ± 0.2	23.4	30.5 ± 0.2	30.5
15	22.6 ± 0.3	22.6	22.4 ± 0.6	22.4	23.4 ± 0.2	23.4	30.5 ± 0.2	30.5

Legend: C1 = stalk from organic management; C2 = stalk from integrated management; C3 = debranning products from integrated management; C4 = debranning products from organic management; exp = experimental; RSM = response surface methodology. Results are expressed as mean ± standard deviation for three replications.

**Table 7:** Analysis of variance for the second-order polynomial equation for ultrasound-assisted extraction of antioxidant activity.

	C1		C2		C3		C4	
	Sum of Square	*p*-Value	Sum of Square	*p*-Value	Sum of Square	*p*-Value	Sum of Square	*p*-Value
X_1_	24.54	<0.0001	36.27	<0.0001	13.20	0.0001	5.98	<0.0001
X_2_	4.29	0.0001	5.21	<0.0001	19.12	<0.0001	2.90	0.0030
X_3_	11.71	<0.0001	1.38	0.0133	0.26	0.5124	2.56	0.0049
X_1_^2^	1.32	0.0160	1.18	0.0201	110.66	<0.0001	4.67	0.0006
X_2_^2^	29.54	<0.0001	2.83	0.0009	8.45	0.0010	23.91	<0.0001
X_3_^2^	7.59	<0.0001	11.00	<0.0001	5.42	0.0060	41.28	<0.0001
X_1_ X_2_	14.06	<0.0001	19.97	<0.0001	0.28	0.4967	0.58	0.1462
X_1_ X_3_	5.58	<0.0001	23.81	<0.0001	1.39	0.1363	51.29	<0.0001
X_2_ X_3_	1.02	0.0315	13.12	<0.0001	0.01	0.9269	17.47	<0.0001
Residual	3.80		3.74		11.50		5.10	
Lack of fit	0.90	0.1925	0.54	0.4324	0.33	0.9153	0.78	0.4066
Pure error	2.90		3.20		11.16		4.32	
Total SS	106.48		120.64		178.62		148.20	
R^2^	0.964		0.969		0.936		0.966	
R_adj_^2^	0.948		0.955		0.907		0.950	
AAD								

Legend: X_1_ = green solvent composition, X_2_ = extraction time, X_3_ = extraction temperature; Legend: C1 = stalk from organic management; C2 = stalk from integrated management; C3 = debranning products from integrated management; C4 = debranning products from organic management.

**Table 8:** Optimal values of UAE process parameters for total phenolic compounds (TPC) and antioxidant activity (AA).

Sample	Response	X_1_ (%)	X_2_ (min)	X_3_ (°C)
C1	TPC	80.0	40	80.0
	AA	50.0	55	80.0
C2	TPC	65.0	60	80.0
	AA	57.5	40	80.0
C3	TPC	80.0	40	77.5
	AA	65.0	40	70.0
C4	TPC	80.0	40	80.0
	AA	80.0	50	77.5

Legend: C1 = stalk from organic management; C2 = stalk from integrated management; C3 = debranning products from integrated management; C4 = debranning products from organic management.

**Table 9:** Comparison between results of ultrasound-assisted extraction (UAE) under optimal conditions and traditional method (TM) for total phenolic compounds (TPC), total flavans content (TFC), chlorophyll a (ChA), chlorophyll b (ChB), yellow pigment (YPG), and antioxidant activity (AA).

Sample	Extraction Method	TPC (mg GAE 100 g^−1^ FW)	TFC (mg CE 100 g^−1^ FW)	ChA (μg 100 g^−1^ FW)	ChB (μg 100 g^−1^ FW)	YPG (g 100 g^−1^ FW)	AA (mg TE 100 g^−1^ FW)
C1	TM	185 ± 1 ^b^	12 ± 1 ^b^	2882 ± 210 ^b^	2493 ± 120 ^b^	2.00 ± 0.02 ^b^	20.5 ± 0.8 ^b^
	UAE	382 ± 3 ^a^	20 ± 2 ^a^	3514 ± 62 ^a^	2899 ± 42 ^a^	6.8 ± 0.3 ^a^	26.0 ± 0.4 ^a^
C2	TM	163 ± 8 ^b^	11 ± 1 ^b^	2012 ± 154 ^b^	1600 ± 85 ^b^	1.60 ± 0.04 ^b^	22.0 ± 1.0 ^b^
	UAE	270 ± 2 ^a^	18 ± 2 ^a^	3510 ± 150 ^a^	2642 ± 165 ^a^	5.8 ± 0.4 ^a^	26.6 ± 0.3 ^a^
C3	TM	203 ± 2 ^b^	1.0 ± 0.1 ^b^	222 ± 12 ^b^	339 ± 22 ^b^	1.78 ± 0.04 ^b^	16.5 ± 0.8 ^b^
	UAE	295 ± 3 ^a^	1.6 ± 0.2 ^a^	459 ± 25 ^a^	606 ± 46 ^a^	1.92 ± 0.03 ^a^	26.1 ± 0.2 ^a^
C4	TM	162 ± 6 ^b^	0.9 ± 0.1 ^b^	323 ± 15 ^b^	425 ± 31 ^b^	2.00 ± 0.05 ^b^	16.4 ± 0.3 ^b^
	UAE	241 ± 2 ^a^	1.5 ± 0.2 ^a^	545 ± 19 ^a^	778 ± 55 ^a^	2.28 ± 0.09 ^a^	27.1 ± 0.3 ^a^

Legend: C1 = stalk from organic management; C2 = stalk from integrated management; C3 = debranning products from integrated management; C4 = debranning products from organic management; FW = fresh weight. Results are expressed as mean ± standard deviation for three replications. Values with different letters in the same parameter are significantly different (*p* ≤ 0.05).

**Table 10:** Comparison of content of individuals phenolic acids (PAs) between conventional and UAE extraction from different byproducts. Data are expressed as mean values ± standard deviation.

Sample	Gallic Acid (μg/mL)	Vanillic Acid (μg/mL)	4-Coumaric Acid (μg/mL)	Ferulic Acid (μg/mL)	Caffeic Acid (μg/mL)	Syringic Acid (μg/mL)	3-Cumaric Acid (μg/mL)	Sinapic Acid (μg/mL)
**C1**								
UAE	14.6 ± 0.9 ^a^	1.72 ± 0.08 ^a^	3.9 ± 0.3 ^a^	3.1 ± 0.1 ^a^	-	-	-	-
TM	tr	tr	0.17 ± 0.02 ^b^	0.52 ± 0.03 ^b^	-	-	-	-
**C2**								
UAE	19.0±1.5 ^a^	1.05 ± 0.01 ^a^	4.1 ± 0.8 ^a^	3.9 ± 0.2 ^a^	-	-	-	-
TM	tr	Tr	0.14 ± 0.03 ^b^	0.59 ± 0.05 ^b^	-	-	-	-
**C3**								
UAE	-	0.10 ± 0.01 ^b^	0.48 ± 0.02 ^a^	0.41 ± 0.06 ^a^	1.84 ± 0.07 ^a^	0.72 ± 0.04 ^a^	0.64 ± 0.03 ^a^	0.10 ± 0.04 ^a^
TM	-	0.20 ± 0.06 ^a^	0.47 ± 0.06 ^a^	0.24 ± 0.05 ^b^	1.67 ± 0.04 ^b^	Tr	0.12 ± 0.03 ^b^	0.18 ± 0.05 ^a^
**C4**								
UAE	-	0.20 ± 0.07 ^a^	0.30 ± 0.05 ^a^	0.6 ± 0.1 ^a^	2.38 ± 0.01 ^a^	0.70 ± 0.10 ^a^	0.97 ± 0.01 ^a^	0.10 ± 0.02 ^a^
TM	-	0.21 ± 0.08 ^a^	0.41 ± 0.02 ^a^	0.3 ± 0.1 ^b^	2.01 ± 0.09 ^b^	0.10 ± 0.01 ^b^	tr	tr

Legend: UAE = ultrasound-assisted extraction; TM = classic extraction technique; tr = trace values; C1 = stalk from organic management; C2 = stalk from integrated management; C3 = debranning products from integrated management; C4 = debranning products from organic management. Results are expressed as mean ± standard deviation for three replications. Values with different letters in the same parameter are significantly different (*p* ≤ 0.05).

**Table 11:** Crumb and crust color characteristics for the three types of bread.

Bread	Crust			Crumb		
	L*	a*	b*	L*	a*	b*
P1	57.27 ± 0.85 ^a^	7.33 ± 1.89 ^a^	28.25 ± 1.55 ^b^	58.33 ± 2.16 ^b^	−6.08 ± 4.75 ^b^	11.34 ± 0.30 ^c^
P2	59.45 ± 1.20 ^a^	5.49 ± 5.03 ^a^	32.34 ± 1.70 ^a^	61.36 ± 2.21 ^ab^	2.15 ± 4.35 ^a^	14.54 ± 0.89 ^b^
P3	56.80 ± 2.41 ^a^	8.72 ± 3.04 ^a^	29.38 ± 1.19 ^b^	62.46 ± 0.89 ^a^	0.48 ± 0.22 ^a^	17.46 ± 1.72 ^a^

Legend: L* = luminosity; a* = redness; b* = yellowness; P1 = control; P2 = bread with 10% of extract; P3 = bread with 20% of extract. Results are expressed as mean ± standard deviation for three replications. Values with different letters in the same parameter are significantly different (*p* ≤ 0.05).

**Table 12:** Total phenolic compounds (TPC) and antioxidant activity (AA) for the three types of bread.

Bread	TPC (mg GAE 100 g^−1^ FW)	AA (mg TE 100 g^−1^ FW)
P1	23.6 ± 0.9 ^c^	3.90 ± 0.09 ^c^
P2	27.2 ± 0.6 ^b^	4.40 ± 0.12 ^b^
P3	30.0 ± 0.8 ^a^	7.00 ± 0.10 ^a^

Legend: P1 = control; P2 = bread with 10% of extract; P3 = bread with 20% of extract; FW = fresh weight. Results are expressed as mean ± standard deviation for three replications. Values with different letters for the same parameter are significantly different (*p* ≤ 0.05).

## Data Availability

The original contributions presented in the study are included in the article; further inquiries can be directed to the corresponding author.
